# VULNERABILITY AND RESILIENCE IN SWISS CENTENARIANS: FINDINGS FROM THE SWISS100 PHONE STUDY

**DOI:** 10.1093/geroni/igad104.0918

**Published:** 2023-12-21

**Authors:** Daniela Jopp, Stefano Cavalli, Francois Herrmann, Armin von Gunten, Mike Martin, Kim Uittenhove, Justine Falciola

**Affiliations:** University of Lausanne, Lausanne, Vaud, Switzerland; University of Applied Sciences and Arts of Southern Switzerland, Manno, Ticino, Switzerland; Geneva University Hospitals, Thônex, Geneve, Switzerland; Lausanne University Hospital, Prilly, Vaud, Switzerland; University of Zurich, Zurich, Zurich, Switzerland; University of Lausanne, Lausanne, Vaud, Switzerland; Geneva University Hospital, Thônex, Geneve, Switzerland

## Abstract

Altfhough every second child born after the year 2000 is expected to become a centenarian, little is known about what characterizes life at age 100. This is also the case for Switzerland, one of the countries with the highest life expectancy world-wide. In this presentation, we will report findings from SWISS100, the first nation-wide centenarian study in Switzerland. During the COVID-19 pandemic, we conducted a telephone study with centenarians and a family member as proxy informant, to investigate specific characteristics and challenges of centenarians. Centenarians were identified through the national address registry. A total of 171 centenarian cases were included in the SWISS100 Phone Study, including reports from 96 centenarians and 102 proxy respondents (mostly children). Centenarians were on average 102 years old, with a range of 100 to 110 years; 75% were women and 25% were men. One third of the centenarians had received basic education only, one third had completed an apprenticeship and about one third had higher education. Most of them lived in institutions (63%). Of those living in private, about half lived alone, one fourth lived with a child, and 12% still lived with a spouse. The majority was widowed. About 85% had children. Over half of the centenarians reported good to excellent subjective health; at the same time, over 70% of the centenarians indicated to often experience health restrictions. Those still able to communicate had high well-being and notable psychological strengths. In sum, findings demonstrate vulnerability but also psychological resilience in Swiss centenarians.

